# Application of a Behavioral Change Approach to a Pediatric Patient Undergoing Transfemoral Amputation

**DOI:** 10.1298/ptr.25-E10348

**Published:** 2026-05-21

**Authors:** Miwa GOTO, Sayaka FUJIWARA, Teruki SHIBATA, Kazuhiko YOKOTA, Mitsuteru HIWATARI, Hiroshi KOBAYASHI, Nobuhiko HAGA

**Affiliations:** 1 Rehabilitation Center, The University of Tokyo Hospital, Japan; 2 TAZAWA MFG. Co. Ltd, Japan; 3 Department of Pediatrics, Teikyo University School of Medicine, Japan; 4 Department of Orthopaedic Surgery, Faculty of Medicine, The University of Tokyo, Japan; 5 National Rehabilitation Center for Persons with Disabilities, Japan

**Keywords:** Transfemoral amputation, Osteosarcoma, Self-efficacy, Self-monitoring, Behavioral change approach

## Abstract

**Background:**

There are no reports on physiotherapy to promote behavioral changes in the immediate postoperative period for lower limb amputees.

**Patient:**

A 7-year-old girl patient underwent transfemoral amputation as a treatment for osteosarcoma located at the proximal fibula.

**Course of Physiotherapy:**

As a result of continuous physiotherapy for the patient from prior to surgery, she was able to obtain a prosthetic gait without support before the end of chemotherapy. The intervention was performed according to the mental state and behavioral change status of this patient, who was reluctant to undergo physiotherapy while undergoing chemotherapy.

**Conclusion:**

In this patient, increasing self-efficacy and improving motivation for prosthetic gait can be attributed to many interventions, such as continuous physiotherapy from before surgery, self-monitoring after surgery, behavioral goal-setting methods appropriate for self-assessment, interaction with other lower limb amputee patients, accumulation of positive experiences, and discussion of collaborative problem-solving methods. The combination of a behavioral change approach in physiotherapy for a pediatric patient undergoing transfemoral amputation was useful.

## Introduction

Physiotherapy for tumor-induced amputations should be provided according to the patient’s general condition, even when performance status is affected by long-term chemotherapy. In pediatric patients, in addition to physical symptoms, psychosocial maladjustments are common, including hospitalization-related stress, illness-related anxiety, decreased self-esteem and self-efficacy, and other psychological issues^1)^. Therefore, addressing mental and psychological aspects by considering the patient’s feelings is essential. Interventions that enhance self-efficacy and promote behavioral change are also necessary for successful prosthetic ambulation. According to the transtheoretical model of behavior change (TTM), individuals progress through five stages—precontemplation, contemplation, preparation, action, and maintenance—when adopting new behaviors^2)^. These stages are not always linear; relapse from maintenance to earlier stages, such as precontemplation, may occur^2)^.

Advancing through the stages requires continuous, individualized interventions, whereas insufficient engagement can hinder behavioral change^2)^. However, no reports to date have described interventions that promote behavioral change in the immediate postoperative period among lower limb amputees.

Here, we present the case of a 7-year-old girl who underwent transfemoral amputation for osteosarcoma of the proximal fibula. Initially resistant to physiotherapy, her motivation improved, and she ultimately achieved independent ambulation with a transfemoral prosthesis. This study was approved by the Ethics Committee of the University of Tokyo (Approval No. 2373-(7)). Written informed consent for this case report was obtained from the patient and family.

## Patient

At 7 years and 10 months of age (day X), the girl developed subjective pain in the left knee. On day X + 2, she presented to a local hospital and was referred to our institution on day X + 6 with a suspected diagnosis of osteosarcoma. An incisional biopsy was performed on day X + 10, confirming conventional osteosarcoma. Although reconstructive surgery was initially planned, the tumor continued to enlarge despite chemotherapy (Fig. 1). Consequently, transfemoral amputation with a stump length of 18 cm was performed on day X + 87.

**Fig. 1.**
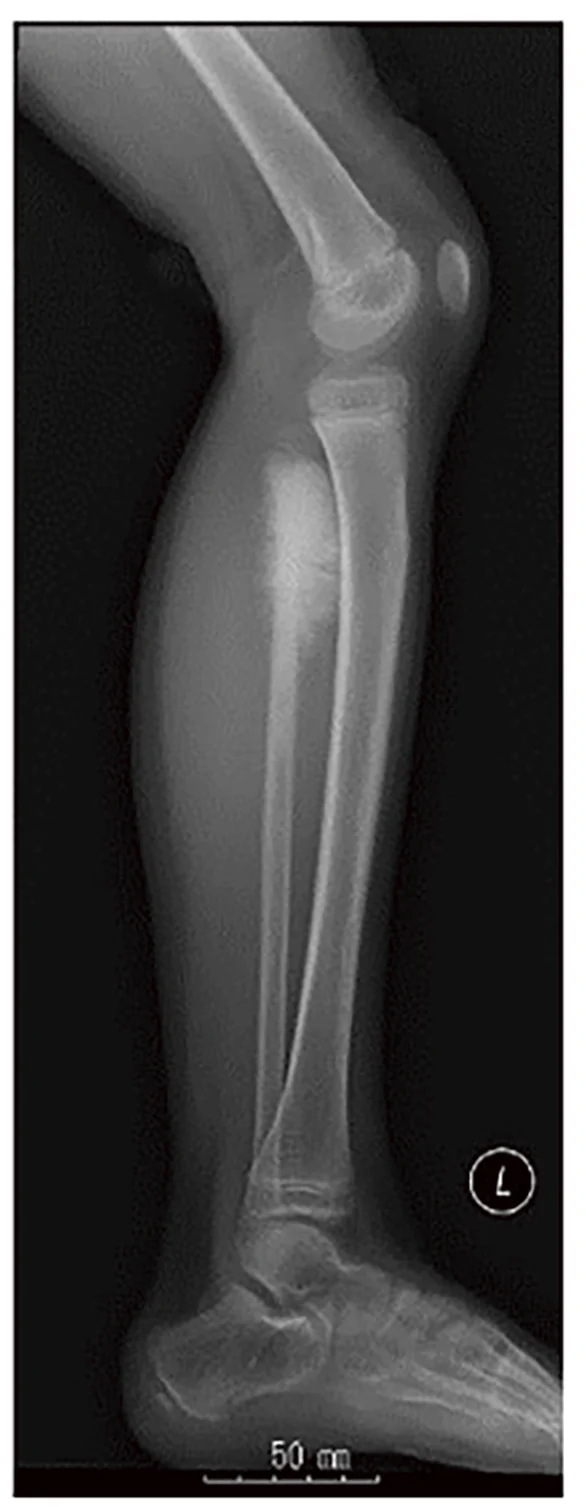
Preoperative imaging findings: Lateral view of her left lower limb on plain X-ray.

## Course of Physiotherapy

### Before amputation

Physiotherapy was initiated on the day of the initial examination at our institution (day X + 6). At that time, swelling of the proximal left lower leg due to the tumor was noted, along with a 35° flexion contracture of the left knee joint. Weight bearing on the affected limb was also limited by pain. The physiotherapy goals at this stage were to improve knee joint contracture, maintain muscle strength, and preserve walking ability. Although contracture management was challenging, knee extension improved to 0° by day X + 54. Ambulation with 2 Lofstrand crutches was achieved immediately after physiotherapy.

Regarding behavioral change, although the patient was afraid of strangers and spoke rarely, communication was possible through non-verbal methods such as nodding. However, she was unable to accept hospitalization or treatment and showed strong resistance—crying and refusing care—for her knee contracture. For her, the use of a cane was regarded not as an acceptance of physiotherapy, but rather as a means of avoiding pain. Based on this behavior, she was assessed to be in a stage between precontemplation and contemplation in the TTM. Because the patient strongly refused any physiotherapy, we tried to establish a relationship of trust by performing exercise therapy centered on play. No self-training was conducted. Introduction of the amputation treatment strategy caused both the patient and the family substantial mental shock. However, as per the family’s wish, preoperative orientation was conducted. This included giving the mother and patient an introduction and explanation of the lower limb prosthesis using the actual prosthesis and showing videos of lower limb amputees running and playing sports using the prostheses (Table 1).

**Table 1.** Patient’s clinical course

	**Before amputation (87 days)**			**Immediately after amputation (30 days)**		**After initiating walking on a prosthetic leg (167 days)**	**After walking independently on a prosthetic leg (196 days)**		
Chemotherapy			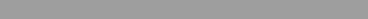			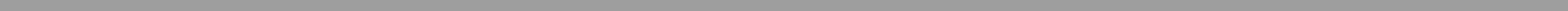			
Rehabilitation process	▲	▲	▲	▲	▲	▲	▲	▲	▲
	First medical examination	Hospitalization	Incisional biopsy	Above-knee amputation	Fitting for a prosthetic leg	Start walking on a prosthetic leg	Walking independently on a prosthetic leg	Walking independently without assistance	Discharge from the hospital
Mental status	Refusal			Reduced refusal Willing to go to school		Benefits outweigh the burden of the prosthetic leg	Willing to walk utilizing the prosthetic leg		
**Stage of behavioral changes**	**Precontemplation to contemplation stage**			**Contemplation to preparation stage**		**Preparation stage**	**Action stage**		
**Approach to behavioral changes**	**Build a relationship of trust**			**Empathize with feelings** **Give a sense of security** **Self-monitoring**		**Goal setting method** **Self-monitoring**	**Activity meters** **Accumulate successful experiences** **Problem-solving method**		
Range of motion exercise	Hip, knee joints			Hip joint		Hip joint	Hip joint		
Muscle strengthening exercise	Hip, knee joints			Muscles around the hip joint		Muscles around the hip joint	Muscles around the hip joint		
Stump, prosthetic leg training	Preoperative orientation			Compression therapy		Wearing the prosthetic leg Weight bearing on the prosthetic leg	Walking on the prosthetic leg Sports		
Prosthetic leg				Temporary prosthetic leg for training: Quadrilateral socket (plug type) Silesian belt Fixed knee joint, SACH foot		Prosthetic leg training: quadrilateral socket Liner pin locking system Fixed knee joint → Polycentric knee joint SACH foot	Temporary prosthetic leg: quadrilateral socket Liner pin locking system Polycentric knee joint, SACH foot		
Walking exercises	Use of 2 Lofstrand crutches			Use of 2 Lofstrand crutches (with prosthetic leg)		Use of 2 Lofstrand crutches (with prosthetic leg)	Use of 2 Lofstrand crutches (with prosthetic leg) → One Lofstrand crutch → walking without assistance		

The detailed procedures during the rehabilitation were changed according to the pediatric patient’s mental state and the behavioral change stage model in 4 stages, that is, “before amputation,” “immediately after amputation,” “after initiating walking on the prosthetic leg,” and “immediately after walking independently on the prosthetic leg.” The intervention included a behavioral change approach in addition to stump management, stump formation, range of motion exercise, muscle strengthening exercise, and walking exercises. Bold values ​​indicate the highlighted results. SACH, solid ankle, cushioned heel

### Immediately after amputation

Postoperative physiotherapy began cautiously. On postoperative day (POD) 3, an attempt to mobilize was unsuccessful because the patient feared increased wound pain with limb movement. Mobilization with two Lofstrand crutches was initiated on POD 5. Phantom limb pain appeared on POD 6, but accurate assessment of its type and severity was difficult due to crying. Sutures were completely removed on POD 10. Compression therapy using a liner was started on POD 13, and mirror therapy for phantom limb pain was introduced on POD 14. Chemotherapy was also resumed on POD 14, and subsequent physiotherapy sessions were adjusted according to the patient’s general condition.

Regarding behavioral change, the patient gradually showed reduced resistance to physiotherapy and began to participate in classroom activities within the hospital. However, she continued to experience strong separation anxiety from her family and had not fully adapted to the hospital environment. Based on her decreasing resistance, she was considered to be between the contemplation and preparation stages in the TTM. Interventions emphasized empathy and reassurance, and she was encouraged to wear the liner daily except at bedtime. Nevertheless, establishing consistent adherence to this behavior remained difficult. Self-monitoring of liner-wearing time was initiated on POD 20, starting at 30 min and aiming to extend the duration by 30 min daily. After initiation, the patient noted a decrease in phantom limb pain related to liner use.

### After initiating walking on the prosthesis

Regarding physiotherapy, on POD 22, wearing the transfemoral prosthesis for training, weight-bearing, and gait exercises were started (Fig. 2C). The socket was a plug-fit socket, and the suspension was a Silesian belt to simplify self-mounting. The knee components were not used to eliminate the fear of falling caused by the knee buckling on the affected side with weight bearing and to encourage a reassuring standing position and gait. The wearing time of the liner was progressively extended, and full-day use was achieved on POD 42. On POD 51, as the piston movement between the stump and socket became an issue, the suspension method was changed to a liner with a locking pin system (Fig. 3). Upon achieving independence in donning the prosthesis during daily activities, the patient began self-monitoring of prosthetic wearing time on POD 72, which permitted the initiation of supervised prosthetic gait training. On POD 119, the prosthesis was completed by changing the knee component to a polycentric knee joint (Total Knee Junior/Össur) because full weight bearing on the prosthesis was achieved. On POD 197, the patient was able to independently walk around the hospital using two Lofstrand crutches.

**Fig. 2.**
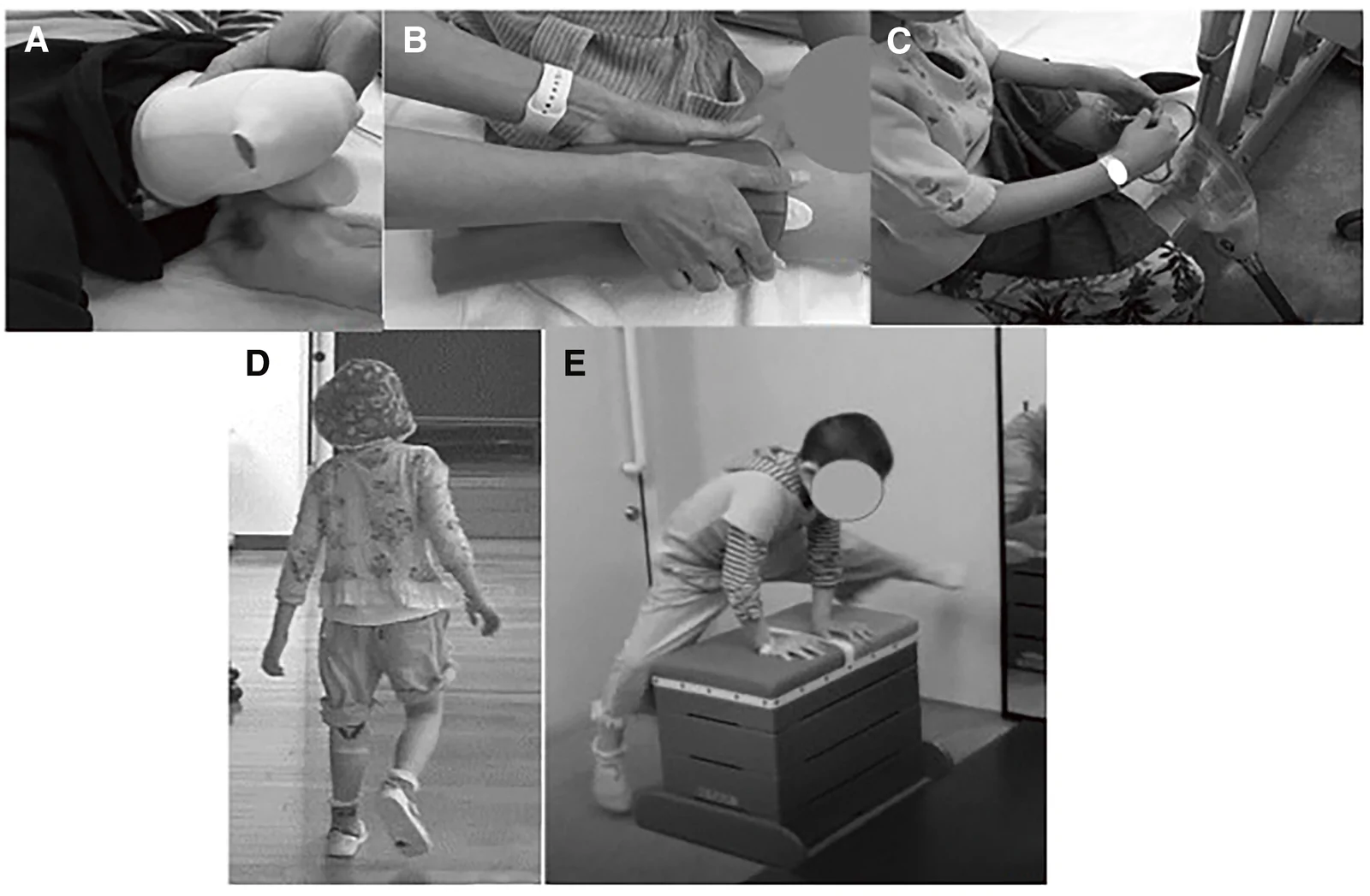
Excerpts of rehabilitation program. (**A**) Compression therapy with elastic bandages: Immediately after amputation (day 12 after surgery). (**B**) Compression therapy with silicone liner: Immediately after amputation (day 13 after surgery). (**C**) Practice wearing the prosthetic leg: After initiating walking on the prosthetic leg (day 51 after surgery). (**D**) Practice walking on the prosthetic leg: After walking independently on the prosthetic leg (day 200 after surgery). (**E**) Exercise therapy using a vaulting horse: After walking independently on the prosthetic leg (day 244 after surgery).

**Fig. 3.**
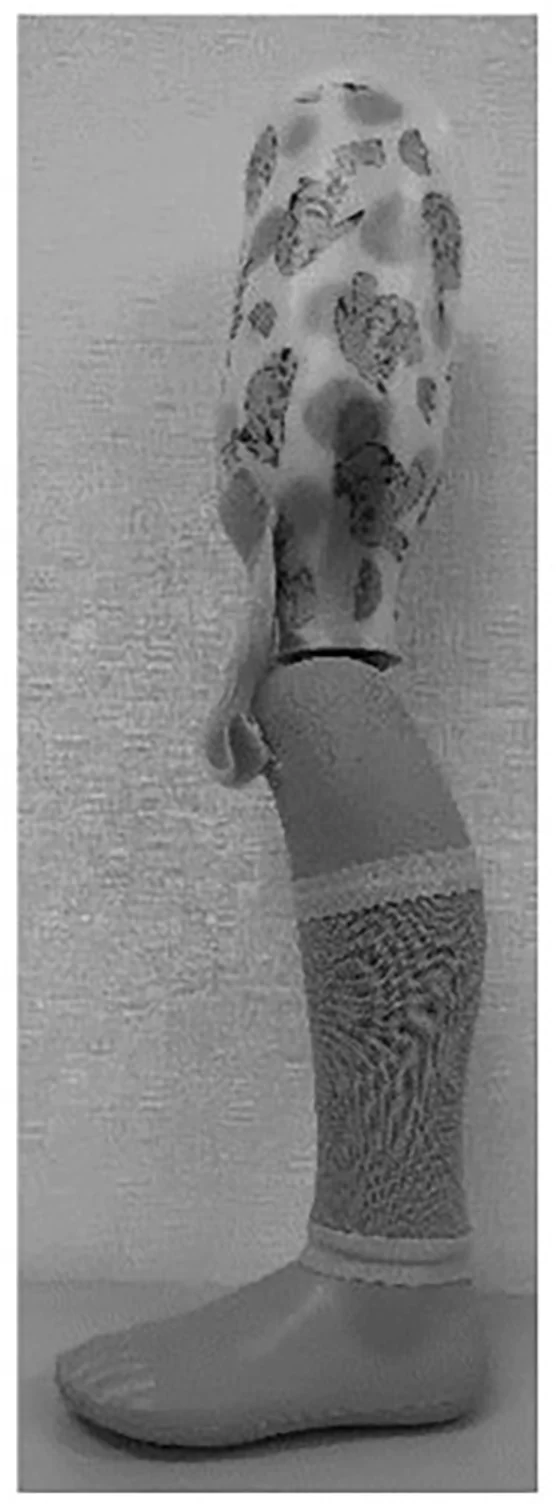
Transfemoral prosthesis (quadrilateral socket, liner with locking pin system, polycentric knee joint, and solid ankle cushioned heel foot).

Regarding behavioral change, the patient gradually recognized the benefits of using a prosthesis, particularly the improvement in phantom limb pain. At this stage, she was assessed to be in the preparation stage of the TTM, as the perceived benefits of wearing the liner and using the prosthesis for gait outweighed the associated burdens. To further improve walking ability, a goal-setting strategy was introduced, allowing the patient to set specific, achievable, and measurable action goals. On POD 27, a peer-support opportunity was arranged in which the patient met an adult transfemoral prosthesis user. Through this interaction, she observed that individuals with transfemoral prostheses could ambulate independently, enjoy activities such as amusement park rides without crutches, and even perform squatting. This experience motivated her to establish the goal of full weight bearing on the prosthesis, which was necessary to transition to a polycentric knee joint. Continuous self-monitoring of liner use enabled the patient to wear the liner consistently throughout the day by POD 41. Despite this progress, she experienced mental stress related to the difficulty and time required for prosthetic fitting, compounded by the unfamiliarity of the family and nursing staff with the procedure and variations in thigh circumference due to chemotherapy. To address this, self-monitoring of prosthesis-wearing time was implemented on POD 72. By POD 83, the patient set functional goals, including walking to her local elementary school without a crutch and avoiding wheelchair use within six months postoperatively. As prosthesis use became habitual, she recognized that prosthetic gait contributed to further reduction in phantom limb pain. These interventions, combining goal setting, peer support, and self-monitoring, facilitated behavioral change, improved adherence to prosthetic use, and promoted functional recovery in this pediatric patient.

### After walking independently on the prosthesis

On POD 163, the patient achieved independent indoor prosthetic ambulation with a cane, and by day 166 was independently ambulating within the ward using a cane.

In the TTM, the patient was assessed to be in the action stage, as she demonstrated a willingness to walk using the prosthesis and was able to consistently incorporate its benefits into her daily life. Progressive functional training was implemented, including freehand walking (POD 139), outdoor and slope walking (POD 156), balloon volleyball (POD 173), single-leg standing with the prosthesis (POD 176), stair practice (POD 187), pedaling on a cycle ergometer (POD 188), walking while dribbling a basketball (POD 201), jumping rope (POD 211), obstacle crossing (POD 232), horizontal bar and vaulting box exercises (POD 244), and initiation of jogging (POD 250). The goal of this program was to improve dynamic balance ability on the prosthesis. Thereafter, a problem-solving method for return to school was discussed with the patient and her family. Specifically, we discussed how much continuous walking distance and what kind of walking ability condition was required to be able to attend school and do after-school practice as soon as possible. Based on the discussion, successful experiences were accumulated by performing long-distance walking, walking up and down a hill, climbing up and down stairs, and practicing walking on unstable ground. In addition, on POD 246, activity monitoring was introduced to increase prosthetic gait opportunities, prevent relapses in behavioral changes, and lead to improvement of self-efficacy. On POD 253, prosthetic gait without using crutches was completely independent. The number of daily steps increased from 4200 on POD 246 to 10000 on day 255. Moreover, the patient participated in a kids’ running race for patients with amputation on POD 298, where she gained experience using a running-specific prosthetic foot. Chemotherapy was completed on POD 370, and the patient was discharged on POD 393 (Fig. 2D and 2E).

To support a smooth transition back to school, a school reintegration support meeting was held with hospital staff (physicians, nurses, physiotherapists, psychologists, child life specialists, and teachers from the hospital school) as well as school personnel (the principal and the homeroom teacher). After discharge, outpatient physiotherapy was provided once or twice per month to prevent functional regression and ensure continuity of physical development. As a result of this support, the patient was able to participate in the school athletic festival two months after discharge.

## Discussion

### Previous studies on gait acquisition in pediatric patients with amputated lower limbs

Few detailed reports have addressed physiotherapy in pediatric patients undergoing lower limb amputation due to tumors^3–6)^. The incidence of osteosarcoma-related amputations has markedly declined over the past decades, from 47% in the 1980s to 9% in the 1990s and 3% in the 2000s^7)^, resulting in a reduced number of recent case reports. Previous studies indicate that the majority of patients require crutch assistance at discharge; for example, Kauzlarić et al. reported that 89.1% of patients used 2 crutches, 4.4% used 1 crutch, and only 6.5% ambulated independently^5)^. Similarly, Nitta et al. described a 12-year-old transfemoral amputee who achieved indoor ambulation with a T-cane at 10 months postoperatively, while using 2 crutches for outdoor ambulation^6)^. In the present case, continuous physiotherapy combined with a behavioral change approach, initiated preoperatively, enabled a 7-year-old girl to achieve independent ambulation without assistance by 8 months postoperatively. These findings suggest that structured, early interventions may facilitate accelerated functional recovery even in younger pediatric patients.

### Importance of self-efficacy in physiotherapy for pediatric patients with amputated lower limbs

Enhancement of self-efficacy is critical for promoting behavioral change^8)^. Self-efficacy refers to an individual’s perception of their capability to perform behaviors necessary to achieve specific outcomes, reflecting confidence in successfully executing required actions^8)^. It is a key psychological determinant of behavioral change and is particularly important in the habituation stage of exercise^8)^. Higher self-efficacy increases the likelihood that an individual will initiate and persist in actions, even in the face of difficulties or failures^8)^. Accordingly, improving self-efficacy is considered essential in physiotherapy for pediatric patients following lower limb amputation, particularly when learning to walk with a prosthesis. Strategies to enhance self-efficacy include direct achievement experiences, vicarious or surrogate experiences, verbal persuasion, and physiological or emotional feedback. The TTM, combined with self-monitoring and goal-setting methods, provides a structured approach for implementing these strategies^2,8–10)^. Consequently, physiotherapy interventions should be tailored to the patient’s individual condition and current stage of behavioral change.

### Intervention methods in this patient

Physiotherapy procedures were adapted according to the patient’s mental state and the TTM across four stages: pre-amputation, immediately post-amputation, after initiating prosthetic walking, and following independent prosthetic ambulation. The TTM, introduced in the early 1980s, guides interventions based on an individual’s readiness or “willingness” to change behavior^2)^, and its application has been widely expanded to various health behaviors. Appropriate strategies differ across stages; thus, interventions must be tailored to the patient’s current stage (Table 2)^10)^. In this case, enhancing self-efficacy for prosthetic gait was the central focus. Before and immediately after amputation, efforts were made to establish trust by empathizing with the patient’s emotions and providing reassurance. Following the initiation of prosthetic gait training, behavioral strategies—including self-monitoring, goal setting, repeated successful experiences, and multidisciplinary collaboration with the patient and her family—facilitated behavioral change and increased motivation to use the prosthesis. Detailed findings and specific interventions are presented in the following sections.

**Table 2.** Stages of behavioral changes based on the transtheoretical model (partially modified from the report by Oka^9)^) and the approach to behavioral change in this patient.

Stage	Precontemplation	Contemplation	Preparation	Action	Maintenance
Assessment classification	No intention to change behavior within 6 months	There is an intention to change behavior within 6 months	There is an intention to change behavior within 1 month	Behavioral changes are being made within 6 months	Behavioral changes have been maintained for at least 6 months and up to 5 years
Characteristics	Does not understand what the problems are (indifference)	Although there is an understanding of the need to change one’s behavior, there is an inability to make decisions and the individual continues on with the same lifestyle	Quite willing to change behavior	Relapse and deviations from desirable behaviors are common	Continues desirable behaviors and there are no large disturbances in everyday life
	Not willing to accept or unwilling to think about it (denial)	Hesitates to start new behaviors or is particular about past behaviors	Changing their behaviors on their own way		
Appropriate approach	Empathize with the implementers’ feelings	Accept the implementers’ current knowledge, thoughts and behaviors	Increase the levels of action in a stepwise manner to reach the desired level of action	Recommend using the help of others	Recommend participating or partaking in local activities
	Talk to the implementer about what they might know and what they could have doubts about, and give participants a sense of security that “if they wanted someone who would understand them, you would be the person to speak with”	Discuss the benefits and burdens of taking action	The implementer decides on specific goals as much as possible by determining the content and time limit when these can be achieved. Provide praise and encourage when the goal is achieved	Encourage the successful use of support from parents, pets, and others who are important in maintaining desirable behaviors	Enable the implementers’ regular participation in local social activities to allow implementers to obtain information on newly available resources
	Provide only the information required by the implementer	Consider the benefits and burdens to others as well as to one’s self, but leave the decision-making to the implementer	Show and have them directly hear what others are doing	Discuss problem solving	Prevent failures from being overlooked
	Do not provide too much information, ascertain the feelings of implementers when providing information			Expect situations of failure and think about failure, and consider ways to deal with these	Adjust implementers’ thoughts and environment that could induce a relapse. Help these individuals to approach the desired behavior as much as possible even if they exhibit relapse
Inappropriate approach	Be pushy	Criticize or assign blame for behaviors that do not change	Understimate the ability of patient who does not reach the desired level of behavior	Be satisfied with behavioral changes that reach the desired target	Be disappointed or assign blame for being unable to accomplish goals
	Provide knowledge in a one-sided manner	Ignore the benefits of continuing to mainly live in a sedentary manner			
	Become argumentative or try to persuade		Provide numerous challenges	Provide challenges one after the other	
Approach to behavioral changes in this patient	Build a relationship of trust	Empathize with feelings	Goal-setting method appropriate for self-assessment	Activity meters	Outpatient physiotherapy to prevent a relapse
	Empathize with the patients’ feelings			Accumulate successful experiences	
	Provide only the information required by the patient and her family	Give a sense of security	Self-monitoring	Discussion of collaborative problem-solving methods	
				Interaction with other lower limb amputee patients	

### Before amputation

The patient was initially assessed to be between the precontemplation and contemplation stages of the TTM due to limited acceptance of hospitalization and treatment, as well as clear refusal of physiotherapy. Persuasive language from authority figures has been reported to enhance self-efficacy^10)^, and encouragement such as “You can do it very well” from attending physicians is considered effective. Accordingly, supportive and affirmative communication from rehabilitation, orthopedic, and pediatric physicians was employed to facilitate positive engagement with physiotherapy in the hospital setting. Despite persistent refusal, inappropriate persuasive techniques were avoided at this stage, and interventions prioritized consideration of the patient’s feelings. Given her mental state, standard preoperative physiotherapy involving active full-body strength training was adjusted. Exercise therapy through play was emphasized to establish trust and reduce anxiety. This approach was considered to have led to the patient leaving her bed earlier and resuming walking practice postoperatively.

### Immediately after amputation

The patient was assessed to be between the contemplation and preparation stages of the transtheoretical model due to a gradual reduction in refusal of physiotherapy. Accordingly, her perspectives and behaviors were respected, while opportunities were provided to discuss the benefits and burdens of physiotherapy and prosthetic gait. A self-monitoring intervention was incorporated into the physiotherapy program, with support from family members and nursing staff. Self-monitoring involves observing or recording one’s behaviors, enabling objective assessment and reinforcement, and has been reported to be effective in various populations, including patients with hypertension^11)^, obesity^12)^, smoking^13)^, diabetes^14)^, ischemic heart disease^15)^, and hemophilia^16)^. In this case, self-monitoring facilitated habituation of liner use. Continuous recording of wearing duration allowed the patient, physiotherapists, nurses, and family to visualize progress, providing real-time feedback that reinforced behavior. Gradual increases in liner-wearing duration were observed, accompanied by favorable outcomes such as reduction in phantom limb pain. This positive feedback further motivated the patient, contributing to stump maturation and the establishment of habitual liner use. Over time, repeated feedback promoted recognition of results, self-regulation, and sustained adherence to the intervention. These findings indicate that structured self-monitoring, combined with individualized support and behavioral stage-appropriate interventions, can enhance patient engagement, promote habituation of prosthetic use, and reinforce functional outcomes in pediatric patients undergoing lower limb amputation.

These interventions were considered to lead to the formation of behaviors that generated self-efficacy and the habituation of wearing liners. Moreover, we persistently monitored the patient until the liner-wearing behavior was established, while also not underestimating the risk of unchanged behavior. As a result, it is believed that the patient’s motivation was prevented from decreasing.

### After initiating walking on the prosthesis

The patient was assessed to be in the preparation stage of the TTM, as the benefits of wearing liners and using the prosthesis for gait outweighed the burdens. In addition, self-monitoring of prosthesis-wearing duration, surrogate experiences, and goal-setting methods were added. Surrogate experiences are used to show successful experiences among patients with similar abilities and problems^10)^. With goal-setting methods, setting clear and specific goals has a higher motivational effect than ambiguous goals. Furthermore, combining them with feedback could result in higher efficacy^17)^. The SMART principle, which emphasizes goals that are Specific, Measurable, Achievable, Relevant, and Time-bound, was applied to physiotherapy planning in a pediatric patient following lower limb amputation. On POD 27, during the initiation of prosthetic gait training, the patient established the first goal: to achieve full weight bearing on the prosthesis as soon as possible to transition from a locked knee joint to a polycentric knee joint. This goal was successfully reached on POD 69. Given the favorable wound condition and steady functional improvement, additional goals were defined on POD 74: (1) to discontinue wheelchair use within 3 months, and (2) to return to the local elementary school after discharge without relying on a cane. Both targets were met, with wheelchair use discontinued on day 201 and school attendance resumed immediately after discharge. These outcomes illustrate the feasibility and effectiveness of SMART-based rehabilitation goal setting in pediatric care. Self-monitoring interventions for prosthesis-wearing duration were also believed to have contributed to the habituation of wearing it. Specifically, the duration of wearing the prosthesis was visualized, results were shared, and feedback was given to the patient, their family, and multiple professionals. In addition, there was a further reduction in phantom limb pain through wearing and walking using the prosthesis. This was considered to contribute to the extension of duration and habituation of wearing the prosthesis. The patient obtained surrogate experiences through peer support with a patient using a transfemoral prosthesis. Specifically, the patient improved self-efficacy for prosthetic gait by listening to the experiences of a peer who is now able to live without using crutches as well as play at the amusement park. Moreover, when the patient was actually able to see that the peer was able to squat even with transfemoral prostheses, she stated that she “wants to change her prosthesis to one where the knees can bend.” This autonomous desire to change to polycentric knee joints led to the patient’s proposed goal of prosthetic gait. The goal-setting method was introduced, and the patient’s feelings were respected and supported to enable the patient to set stepwise goals on their own. The content and status of goal achievement were shared with the patient, the family, and multiple professionals. Specifically, praise was given in recognition of the patient’s daily living activities, the status of achieving her goals, and the experience of achieving past goals. Experiences of achievement that have been shown to be effective in improving self-efficacy refer to performing tasks according to the patient’s abilities and experiencing successful experiences numerous times^11)^. In this patient, the experience of achieving improvements in independent walking and no longer having to use a wheelchair earlier than planned were considered to result in physiological and emotional enhancement, which led to further improvements in self-efficacy.

### Immediately after walking independently on the prosthesis

In the TTM, the patient was assessed to be in the action stage, as she demonstrated a willingness to walk using the prosthesis and was able to consistently incorporate its benefits into her daily life. A problem-solving method, experiences with achievement, and wearing activity meters were introduced with the goal of preventing dropout from prosthetic gait, relapse of behavioral changes, and improving self-efficacy. Regarding the problem-solving method, the amount of endurance and movement ability necessary to be discharged home and return to school was carefully discussed with the patient and the family. To provide the achievement experiences, we made her successful experiences include walking long distances without crutches, walking outdoors on unstable ground such as hills and bad roads, and playing sports. These provided her the confidence to return to daily life and school wearing the prosthesis, as well as an improvement in self-efficacy. These approaches also led to her being able to walk independently before the completion of chemotherapy. Gaining an understanding of the quantity of one’s activity through wearing activity meters provided the patient with feedback, such as information on the extent of prosthetic gait. This knowledge could prevent adverse events, such as stump pain and fatigue, which then led to improving the patient’s motivation for using the prosthesis.

## Conclusions

Continuous physiotherapy with a behavioral change approach was performed preoperatively for a pediatric patient who underwent transfemoral amputation because of osteosarcoma. Independent prosthetic gait without assistance was achieved during chemotherapy administration. Regarding behavioral change approaches, the key focus was on improving the patient’s self-efficacy for walking with the prosthesis. The approaches tailored to each stage of behavioral change included building trust (precontemplation–contemplation: before and immediately after amputation), providing empathy and a sense of security (contemplation: immediately after amputation), setting goals and implementing self-monitoring (preparation: after initiating prosthetic gait training), and employing activity meters, reinforcing achievements, and applying problem-solving strategies (action: immediately after achieving independent ambulation with the prosthesis). Collectively, these interventions facilitated behavioral change and enhanced motivation to use the prosthesis. These interventions resulted in the ability to walk independently without assistance at an early stage. In this patient of school age, self-monitoring and goal-setting methods were considered useful for improving motivation and behavior change toward prosthetic gait.

### Limitation of this study

Since the girl was 7 years old, it was not feasible to assess self-efficacy and mental state using quantitative scales. These scales include the Self-Efficacy Scale, Exercise Self-Efficacy Scale, Behavioral Regulation in Exercise Questionnaire, and Treatment Self-Regulation Questionnaire.

## References

[1] Stanton, AL, Collins, CA, et al.: Adjustment to chronic illness: theory and research. In: AS Baum, TA Revenson, et al.: Handbook of Health Psychology. 1st ed, Lawrence Erlbaum Associates, New Jersey, 2001, 387–403.

[2] Procaska JO, DiClemente CC: Transtheoretical therapy: toward a more integrative model of change. Psychother (Chic). 1982; 19: 276–288.

[3] Cole WG, Klein RW, et al.: Prosthetic programme after above-knee amputation in children with sarcomata. J Bone Joint Surg Br. 1982; 64-B: 586–589. 10.1302/0301-620X.64B5.6958675 6958675

[4] Murphy AD, Atkins SE, et al.: The use of vascularised bone capping to prevent and treat amputation stump spiking in the paediatric population. Microsurgery. 2017; 37: 589–595. 10.1002/micr.30160 28121366

[5] Kauzlarić N, Kauzlarić KS, et al.: Prosthetic rehabilitation of persons with lower limb amputations due to tumour. Eur J Cancer Care (Engl). 2007; 16: 238–243. 10.1111/j.1365-2354.2006.00727.x 17508943

[6] Nitta K, Kodama M, et al.: Prosthetic rehabilitation for a pediatric patient with trans-femora amputation due to osteosarcoma during inpatient chemotherapy: A case report. J Clin Rehabil. 2018; 27: 494–497. [in Japanese]

[7] Ayerza MA, Farfalli GL, et al.: Does increased rate of limb-sparing surgery affect survival in osteosarcoma? Clin Orthop Relat Res. 2010; 468: 2854–2859. 10.1007/s11999-010-1423-4 PMC2947695 20559766

[8] Bandura A: Self-efficacy: Toward a unifying theory of behavioral change. Psychol Rev. 1977; 84: 191–215. 10.1037//0033-295x.84.2.191 847061

[9] Oka K: Recent trends of research on exercise adherence utilizing the transtheoretical model of behavior change. Jpn J Phys Educ Hlth Sport Sci. 2000; 45: 543–561. [in Japanese]

[10] Bandura A: Social foundations of thought and action: a social cognitive theory. Prentice-Hall, Ney Jersey, 1986.

[11] Carnahan JE, Nugent CA: The effects of self-monitoring by patients on the control of hypertension. Am J Med Sci. 1975; 269: 69–73. 10.1097/00000441-197501000-00008 1130437

[12] Wing RR, Hill JO: Successful weight loss maintenance. Annu Rev Nutr. 2001; 21: 323–341. 10.1146/annurev.nutr.21.1.323 11375440

[13] Israel AC, Raskin PA, et al.: The effects of self-monitoring of smoking and awareness of accuracy feedback upon a concurrent behavior. Addict Behav. 1979; 4: 199–203. 10.1016/0306-4603(79)90055-8 474270

[14] Worth R, Home PD, et al.: Intensive attention improves glycaemic control in insulin-dependent diabetes without further advantage from home blood glucose monitoring: results of a controlled trial. BMJ. 1982; 285: 1233–1240. 10.1136/bmj.285.6350.1233 PMC1499792 6812820

[15] Izawa KP, Watanabe S, et al.: Effect of the self-monitoring approach on exercise maintenance during cardiac rehabilitation: a randomized, controlled trial. Am J Phys Med Rehabil. 2005; 84: 313–321. 10.1097/01.phm.0000156901.95289.09 15829777

[16] Goto M, Takedani H, et al.: Self-monitoring has potential for home exercise programmes in patients with haemophilia. Haemophilia. 2014; 20: e121–e127. 10.1111/hae.12355 24418413

[17] Locke EA: Toward a theory of task motivation and incentives. Organ Behav Hum Perform. 1968; 3: 157–189.

